# Associated Factors and Prognostic Implications of Non-convulsive Status Epilepticus in Ischemic Stroke Patients With Impaired Consciousness

**DOI:** 10.3389/fneur.2021.795076

**Published:** 2022-01-07

**Authors:** Liren Zhang, Wensi Zheng, Feng Chen, Xiaolin Bai, Lixia Xue, Mengke Liang, Zhi Geng

**Affiliations:** ^1^Department of Neurology, Shanghai Jiao Tong University Affiliated Sixth People's Hospital, Shanghai, China; ^2^Shanghai Key Laboratory of Psychotic Disorders, Department of Psychiatry, Shanghai Mental Health Center, School of Medicine, Shanghai Jiao Tong University, Shanghai, China; ^3^Department of Neurology, Shanghai Jiao Tong University Affiliated Sixth People's Hospital South Campus, Shanghai, China

**Keywords:** non-convulsive status epilepticus, cerebral ischemic stroke, outcome, consciousness disorder, neuro intensive care unit

## Abstract

**Background and Purpose:** Non-convulsive status epilepticus (NCSE) is common in patients with disorders of consciousness and can cause secondary brain injury. Our study aimed to explore the determinants and prognostic significance of NCSE in stroke patients with impaired consciousness.

**Method:** Consecutive ischemic stroke patients with impaired consciousness who were admitted to a neuro intensive care unit were enrolled for this study. Univariate and multivariable logistic regression were used to identify factors associated with NCSE and their correlation with prognosis.

**Results:** Among the 80 patients studied, 20 (25%) died during hospitalization, and 51 (63.75%) had unfavorable outcomes at the 3-month follow-up. A total of 31 patients (38.75%) developed NCSE during 24-h electroencephalogram (EEG) monitoring. Logistic regression revealed that NCSE was significantly associated with an increased risk of death during hospital stay and adverse outcomes at the 3-month follow-up. Patients with stroke involving the cerebral cortex or those who had a severely depressed level of consciousness were more prone to epileptogenesis after stroke.

**Conclusion:** Our results suggest that NCSE is a common complication of ischemic stroke, and is associated with both in-hospital mortality and dependency at the 3-month follow-up. Long-term video EEG monitoring of stroke patients is, therefore required, especially for those with severe consciousness disorders (stupor or coma) or cortical injury.

## Introduction

An increasing number of new electroencephalogram (EEG) patterns have been discovered and have received extensive attention from researchers in the past few years owing to the extended monitoring periods and rapid development of EEG hardware equipment. Non-convulsive status epilepticus (NCSE) can be missed during short-term EEG recordings due to the limited sampling time (<30 min). However, NCSE measurement may be critical in stroke patients and could serve as an EEG marker with prognostic and predictive value. The negative effect of NCSE on patient outcomes has been gradually confirmed by clinical studies in patients with head trauma or cardiac arrest (1, 2). However, little is currently known about its clinical significance in patients with stroke. Meanwhile, it is unclear as to when, how, and under what conditions pharmacological interventions should be delivered due to the absence of noticeable symptoms of NCSE (3). Therefore, it is imperative to understand the predictors and prognostic significance of NCSE. The available evidence on its prognostic value in stroke patients is limited and conflicting (4). This study aimed to examine the prognostic significance of NCSE in stroke patients with altered consciousness and explored possible risk factors.

## Materials and Methods

This was a single-center observational study of stroke patients with disorders of consciousness. The local ethics committee of the Shanghai Jiaotong University Affiliated Sixth People's Hospital approved this study, and informed consent was obtained from the patients' family members.

### Study Population

In this study, 80 consecutive patients with disorders of consciousness were admitted to the NICU at the Shanghai Jiaotong University Affiliated Sixth People's Hospital between June 2020 and May 2021 with acute anterior circulation ischemic stroke. The inclusion criteria were as follows: (1) acute ischemic stroke accompanied by neurological symptoms diagnosed by brain CT or MRI; (2) varying degrees of consciousness disturbance (Glasgow Coma Score [GCS] ≤14), ranging from lethargy to coma (Reaction Level Scale [RLS] ≥2); (3) age >45 years and patient was previously independent (premorbid score of mRS <1).

The exclusion criteria were as follows: (1) pre-existing disorders of consciousness or cognitive impairment; (2) history or family history of epilepsy; (3) other medical conditions that may severely impact electrical activity, such as traumatic brain injury, intracranial tumors, intracranial infection, mental illness, and mechanical ventilation within 48 h of admission; (4) severe comorbidities or other significant systemic diseases; (5) death within 72 h of hospitalization; (6) absence of a written informed consent or refusal to participate in the study.

### Anthropometric and Laboratory Measurements

The following data were collected within 24 h of admission using an electronic medical history system: age, sex, hyper-early treatment, cortical infarcts, hemorrhagic transformation, location of the new foci, body mass index (BMI), HbA1c, low-density lipoprotein, homocysteine, creatinine, urea, uric acid, neuron-specific enolase and history of atrial fibrillation, hypertension, and smoking. Hyper-early treatment refers to thrombolysis or mechanical thrombectomy. The Glasgow Coma Scale (GCS) score and the state of consciousness of the patients were assessed by a professional researcher at the beginning of the EEG examinations. We then categorized GCS into the low-score group (GCS score ≤8) and high-score group (GCS score >8), while the degree of consciousness disorder was divided into three grades: lethargy (RLS = 2), stupor (RLS = 3), or coma (RLS = 4–8).

### EEG Recording

The enrolled patients, managed by experienced neurological physicians who were blinded to the details of the study, were treated according to the guidelines for the diagnosis and treatment of acute ischemic stroke in China. Continuous video-EEG monitoring was performed (as early as possible) within 48 h after admission to the neuro intensive care unit (NICU) and patients were monitored continuously for 24 h, with EEG readouts displayed on their bedside monitor (Xltek, Canada). Signals were obtained using 11 standard 9-mm disc electrodes (eight recording channels: Fp1, Fp2, C3, C4, O1, O2, T3, and T4, two reference electrodes (A1 and A2) and one ground electrode (Fz), which were arranged according to the international 10–20 system. The EEG amplifier parameters were as follows: a time constant of 0.30 s, sensitivity of 7 μV/mm, and low- and high-pass filters were set at 0.3 and 35 Hz, respectively (5, 6). The EEG artifacts were corrected by a professional electrophysiological technician every 4–8 h. NCSE was independently diagnosed according to the Salzburg criteria (7) by two experienced electrophysiologists who were blinded to the clinical information. Any disagreements between specialists were resolved by consensus. Currently, there are no guidelines for active drug intervention for NCSE caused by stroke. Patients experiencing NCSE during the 24-h EEG monitoring were not treated with antiepileptic drugs.

### Outcome Measurement

The primary objective was to investigate the correlation between NCSE and in-hospital death as well as poor outcome at the 3-month follow-up. In addition, we analyzed several clinical characteristics of the patients using regression analysis to determine the risk factors associated with the occurrence of NCSE. A poor outcome was defined as a modified Rankin Scale (mRS) score >3 at the 3-month follow-up after stroke onset.

### Statistical Analysis

All statistical analyses were performed using SPSS, version 25.0 (IBM Corp, Armonk, NY, USA). For descriptive statistics, data with a normal distribution were expressed as mean ± standard deviation, continuous data with non-normal distribution were expressed as median (interquartile range), and categorical variables were expressed as frequency (percentage). We compared baseline characteristics between patients with and without NCSE using the Pearson chi-squared test or Fisher's exact test for categorical variables and Student's *t*-test or Mann–Whitney U test for continuous variables.

We performed logistic regression analysis to identify the risk factors for NCSE as well as the prognostic value of the outcome. A multivariable logistic regression analysis was performed on the factors that were revealed to be statistically significant by univariate analysis to control for the possible effect of confounders. Results are reported as odds ratios (ORs) and corresponding 95% confidence intervals (CIs). Differences were considered statistically significant at *P* < 0.05.

## Results

NCSE was common during the monitoring period in patients admitted to the NICU with acute schemic stroke. In this study, NCSE was observed in 38.75% of the patients, which was probably due to the cohort design (inclusion of patients with a reduced level of consciousness). Eighty patients (36 men and 44 women) enrolled in this study had varying degrees of consciousness disturbance, including 32 cases of lethargy, 24 cases of stupor, and 24 cases of coma. Of these, 51 patients received hyper-early treatment, and 31 patients were assigned to the low-score group. Detailed clinical information of the cohort is presented in Table 1.

**Table 1:** Comparison between NCSE group and Non-NCSE group.

**Variables**	**All (*n* = 80)**	**NCSE (*n* = 31)**	**Non-NCSE (*n* = 49)**	***P*-value**
Age, median (IQR)	78.5 (66.0,87.0)	83 (74.0,87.0)	76 (64.5–86.5)	0.148
Female, *n* (%)	44 (55.0%)	21 (67.7%)	23 (46.9%)	0.068
Hyper-early treatment, *n* (%)	51 (63.8%)	17 (54.8%)	34 (69.4%)	0.187
Low GCS score, *n* (%)	33 (41.3%)	18 (58.1%)	15 (30.6%)	0.015
State of consciousness				0.008
Lethargy, *n* (%)	32 (40.0%)	6 (19.4%)	26 (53.1%)	
Stupor, *n* (%)	24 (30.0%)	11 (35.5%)	13 (26.5%)	
Coma, *n* (%)	24 (30.0%)	14 (45.2%)	10 (20.4%)	
Location of foci				0.724
Left, *n* (%)	22 (27.5%)	10 (32.3%)	12 (24.5%)	
Right, *n* (%)	43 (53.8%)	16 (51.6%)	27 (55.1%)	
Bilateral, *n* (%)	15 (18.8%)	5 (16.1%)	10 (20.4%)	
Hemorrhagic transformation, *n* (%)	21 (26.3%)	11 (35.5%)	10 (20.4%)	0.135
Cortical injury, *n* (%)	49 (61.3%)	24 (77.4%)	25 (51.0%)	0.018
Atrial fibrillation, *n* (%)	43 (53.8%)	18 (58.1%)	25 (51.0%)	0.538
Diabetes, *n* (%)	25 (31.3%)	10 (32.3%)	15 (30.6%)	0.877
Hypertension, *n* (%)	61 (76.3%)	24 (77.4%)	37 (75.5%)	0.854
Smoking, *n* (%)	12 (15.0%)	2 (6.5%)	10 (20.4%)	0.167
BMI, mean ± SD	24.2 ± 4.0	23.6 ± 4.3	24.7 ± 3.8	0.204
hBA1C, median (IQR)	5.8 (5.4, 6.3)	5.7 (5.4,6.2)	5.8 (5.5,6.5)	0.181
Low-density lipoprotein, mean ± SD	2.8 ± 1.1	2.7 ± 0.9	2.8 ± 1.2	0.547
Homocysteine, median (IQR)	15.7 (11.8, 20.5)	17.1 (11.9,23.7)	14.9 (11.5,19.8)	0.432
Creatinine, median (IQR)	82.3 (61.2, 103.8)	83.0 (61.0,107.3)	80.6 (62.0,94.4)	0.671
Urea, median (IQR)	6.3 (4.7, 8.4)	7.1 (4.5,8.4)	6.1 (4.7,8.3)	0.898
Uric acid, mean ± SD	369.3 ± 130.6369	362.9 ± 134.8	373.3 ± 129.2	0.949
Neuron-specific enolase, median (IQR)	16.8 (14.0, 23.6)	17.8 (14.1,35.0)	16.4 (13.9,21.0)	0.189

*NCSE, Non-convulsive status epilepticus; BMI, Body mass index; HbA1c, glycosylated hemoglobin*.

In comparison to the subgroup without NCSE, those who were diagnosed with this complication showed a significant difference in GCS score (*P* = 0.015), state of consciousness (*P* = 0.008), and cortical injury (*P* = 0.018). The clinical indices with statistical significance (identified in Table 1) were included in a multivariable logistic regression analysis, which showed that severe disturbance of consciousness (stupor or coma) and cortical injury are independent risk factors for NCSE after adjusting for confounding factors (Table 2).

**Table 2:** Multivariable logistic regression results of NCSE.

	**OR**	**95% CI**	***p*-value**
State of consciousness (coma)			0.017
Stupor	0.749	0.226–2.480	0.636
Lethargy	0.183	0.053–0.630	0.007
Cortical injury	3.174	1.079–9.336	0.036

*OR, odds ratio; 95% CI, 95% confidence interval*.

In the follow-up results of the study, the overall prognosis of patients was not satisfactory, whereas 29 (36.25%) patients had a good prognosis signature. Twenty-five patients died during the study duration; 20 deaths occurred during hospitalization. The pre-disposing risk factors for in-hospital death included NCSE (*P* < 0.001), age (*P* = 0.036), low GCS score (*P* = 0.013), state of consciousness (*P* =0.003), and neuron-specific enolase (*P* =0.005). After controlling for potential confounders, the analysis revealed that NCSE (OR: 4.916, CI: 1.415–17.078, *P* = 0.012) and neuron-specific enolase (OR: 1.059, CI: 1.007–1.113, *P* = 0.025) were predictors of in-hospital mortality (Table 3).

**Table 3:** Logistic regression results of in-hospital mortality.

**Variables**	**Died (*n* = 20)**	**Non-died (*n* = 60)**	**Crude *P*-value**	**Adjusted OR**	**Adjusted 95% CI**	**Adjusted *P*-value**
NCSE, *n* (%)	14 (70.0%)	17 (28.3%)	<0.001	4.916	1.415–17.078	0.012
Neuron-specific enolase, median (IQR)	29.2 (14.9,53.6)	16.3 (13.9,20.8)	0.005	1.059	1.007–1.113	0.025
Age, median (IQR)	84.5 (76.3,88.5)	77.0 (65.3,86.8)	0.036	/	/	0.346
Low GCS score, *n* (%)	13 (65.0%)	20 (33.3%)	0.013	/	/	0.342
State of consciousness,	/	/	0.003	/	/	0.308
Coma, *n* (%)	12 (60.0%)	12 (20.0%)	/	/	/	/
Stupor, *n* (%)	4 (20%)	20 (33.3%)	/	/	/	/
Lethargy, *n* (%)	4 (20%)	28 (46.7%)	/	/	/	/

*NCSE, Non-convulsive status epilepticus*.

Multiple factors, including NCSE, age, low GCS score, state of consciousness, cortical involvement, atrial fibrillation, neuron-specific enolase are associated with a poor prognosis on univariate analyses. On multivariable analysis, age (OR: 1.126, CI: 1.057–1.199, *P* < 0.003) and low GCS (OR: 7.652, CI: 1.699–34.463, *P* = 0.008) score retained a significant association with the trend for the less favorable disability outcomes at 3- month follow-up. Likewise, NCSE was independently associated with unfavorable prognosis in either unadjusted or adjusted (OR: 12.142, CI: 2.348–62.800, *P* = 0.003) regression models (Table 4).

**Table 4:** Independent predictors of poor outcome at three-months follow-up.

	**OR**	**95% CI**	***P*-value**
NCSE	12.142	2.348–62.800	0.003
Age	1.126	1.057–1.199	<0.001
Low GCS score	7.652	1.699–34.463	0.008

*NCSE, Non-convulsive status epilepticus; OR, odds ratio; 95%CI, 95% confidence interval*.

## Discussion

While the exact incidence and prevalence remain unclear, NCSE is probably a common comorbidity in stroke patients, and its true incidence may be underestimated in the current report since it is underdiagnosed and often mistaken for other disorders (8). Stroke itself seems to be associated with a heightened risk of NCSE, with previous studies confirming stroke as the main cause of NCSE in their cohort (9). The overall prevalence of NCSE in our study population was 38.75%, which is in line with occurrence rates reported in previous studies that range from 14 to 45.3% (10–13). We likewise found that there was a greater rate of NCSE in our cohort than in exclusively retrospective studies. This might be attributed to the following reasons: first, the possibility of a biased selection of study subjects, as many patients with NCSE may not have undergone EEG examinations in the retrospective study, particularly those with no obvious clinical manifestations. The higher median age of our patients may also explain the higher prevalence of NCSE. Recent studies have found that NCSE typically occurs in ICU patients, especially in the elderly and the critically-ill patients (14, 15). Moreover, the subjects analyzed in our study presented with altered consciousness, which may be one of the reasons for the high NCSE occurrence observed in the study. It is generally agreed that altered consciousness is an important risk factor for the development of NCSE, as demonstrated in this study, while others believe that NCSE is the cause of the decreased level of consciousness (11, 16–18). Therefore, while evaluating patients presenting with an altered consciousness, it is important to consider the possibility of NCSE, particularly in those who do not respond to standard treatment. However, the causal relationship between NCSE and patient consciousness remains controversial and requires further research (19, 20).

To our knowledge, this is the first study to report cortical damage as an independent risk factor for NCSE. While it has long been recognized that ischemia of the cortex may be one of the important mechanisms for the generation of epileptiform activity and convulsive epilepsies, impairments in cortical activity have not been known to promote the onset of non-convulsive seizures (21). Recent studies have, however, provided additional evidence to support the correlations between them. One reason for this might be the imbalance of excitatory and inhibitory neurotransmission caused by avascular necrosis in the cortex, leading to abnormally increased excitability in the cortical region (22). Patients with persistent seizures can benefit significantly from the resection of the adjacent epileptogenic cortex (23). Evidence from animal experiments further confirms that some seizures initially originate in local cortical regions before spreading to other regions of the brain (24). Tomari and Belcastro also claim that cardioembolic stroke or large artery atherothrombosis tends to cause cell death in the cortex, and these may be underappreciated risk factors for NCSE; stroke caused by these two factors render the cortex susceptible to epileptic seizures (25, 26). We did not observe a higher risk of seizures in patients with atrial fibrillation, which may be attributed to the availability of aggressive therapeutic strategies, including intravenous thrombolysis and mechanical thrombectomy. The proportions of patients receiving these two specific therapeutic modalities across studies has been variable, with few reaching as high as 64%, as in our study.

Focal cortical irritation caused by blood metabolites in patients with intracerebral hemorrhage may predispose them to acute non-convulsive seizures (27). However, whether hemorrhagic transformation promotes seizures remains controversial. The main reason for this discrepancy could be the small size of bleeding relative to lobar hemorrhages and the distance of the cerebral cortex from the bleeding site. No significant correlations were found for these two parameters in our study, and additional studies are needed to explore the exact clinical relevance and possible mechanisms of NCSE.

Overall, the patients included in our study had a poor prognosis, with a high in-hospital mortality rate that could be attributed to the high proportion of severely ill patients included in the study after the exclusion of mild patients. All patients were admitted to the NICU and experienced different degrees of consciousness disorders. Several previous studies have found potential links between NCSE and poor stroke outcomes and reported mortality rates ranging from 11.9 to 51% (28–31). Additionally, advanced age is a well-known independent factor adversely affecting the prognosis of stroke patients (32), which was confirmed in our study. Compared to previously studied cohorts, the patients in our study were older and the median patient age was 78.5 years with an interquartile range of 66.0–87.0.

We identified several significant prognostic factors and our findings show that a higher neuron-specific enolase level correlated with higher in-hospital mortality, whereas other indices, including age and low GCS score, were independent risk factors that independently influenced patient independence at the 3-month follow-up. Only NCSE was correlated with both measures of outcome; therefore, it may be considered a detection index for lower survival rate and poor prognosis.

Shneker et al. observed that NCSE in patients correlated with substantial mortality (18%) and morbidity (39%) as early as 2003 (33). Patients diagnosed with this condition have longer hospital stays and experience more comorbid illnesses (10, 34). Similarly, Kikuta et al. showed that compared to the subgroup without NCSE, the risk of unfavorable outcomes at 3-months was nearly five-fold higher in patients with aneurysmal subarachnoid hemorrhage (34). The existing evidence in critically-ill patients suggests that NCSE may contribute to secondary brain injury or perpetual nerve damage. These patterns correlate with increased brain metabolism, similar to that observed in patients with convulsive epilepsies (35). This phenomenon was confirmed in some positron-emission tomography studies that revealed a higher level of metabolism in the discharge area (36). Intracranial multi-modal depth monitoring in patients with subarachnoid hemorrhage provides indirect evidence of elevated metabolic activity with the generation of discharge activity, which includes brain tissue oxygenation reduction, increased release of brain glucose, and increased cerebral lactate/pyruvate ratio (35, 37). Inadequately matched hypermetabolism should be considered as one of the causes of neurological deterioration in patients with epileptiform discharges. A recent study by Scoppettuolo et al. found that non-convulsive epileptic activity has a negative impact on the rehabilitation process after stroke. Neurological deterioration caused by NCSE is, therefore, an extremely important factor contributing to poor recovery (16).

Paul et al. also found that the duration and frequency of discharges are key influencing factors during metabolic crisis development and could worsen patient prognosis. Both seizure duration and delayed diagnosis of NCSE have been reported to be significantly associated with increased mortality in severely-ill patients. Early detection and timely intervention for secondary neural dysfunction induced by NCSE is a cornerstone in the management of neurocritically ill patients (26, 28). Although much of the data are from patients with subarachnoid hemorrhage and traumatic brain injury, the hypermetabolism associated with NCSE has been seen across multiple disease etiologies, including ischemic stroke (28). Tabaeizadeh et al. observed a positive dose-dependent association between seizure burden and worse outcomes in patients with stroke. Similarly, a study from Thailand demonstrated that every 1-h increment in the discharge duration was associated with a 1.10-fold [95% (CI) 1.01–1.21, *p* = 0.04] increased risk of unfavorable outcomes and a 1.16-fold [95% (CI) −0.33–0.05, *p* = 0.01] decrease in the TICS-score (30, 38, 39). Longer duration and accelerated frequency of abnormal brainwaves breaks the fragile balance between metabolite need and consumption in the ischemic area, and timely intervention can effectively alleviate the adverse metabolic disturbances of seizure activity in fragile brain tissue. This conclusion was supported by animal experiments. Levetiracetam can improve ischemic brain injury in rats with middle cerebral artery occlusion by suppressing non-convulsive seizure activity (40). Some researchers propose that NCSE itself is probably an important manifestation of severe neurological deficits, which can induce significantly elevated levels of neuron-specific enolase (NSE), a marker of acute brain injury (5, 41, 42). Similar correlations were found in our results, and this causal relationship requires further verification.

Whether these study results can be generalized in stroke patients remains to be clarified because there are studies that have reported contrasting data (9). A small-scale prospective cohort study revealed that the overall prognosis in patients diagnosed with NCSE was not significantly different from those without this complication (29, 43, 44). The findings in another study that included 145 ICU patients indicated that NCSE is a common complication in patients with altered mental status, and both patient outcome and hospital stay did not differ significantly between patients with and without NCSE. The use of antiepileptic drugs may explain this discrepancy (12). On the other hand, some scholars also believe that the different inclusion criteria for patients might yield different results, and etiology is the most important factor influencing prognosis.

From the perspective of the current challenges in managing critically-ill stroke patients, our results have important practical implications for clinical decision-makers. This research not only confirmed a significant correlation between NCSE and poor clinical outcomes but also analyzed factors associated with NCSE occurrence, including serious deterioration of consciousness and cortical injury.

### Limitations and Future Perspectives

Our study has several limitations: (1) The small sample size and single-center data fails to represent all the characteristics of stroke patients and limited the generalizability of the conclusions, and therefore, the conclusions need further confirmation by additional multi-center studies enrolling sufficient number of patients. (2) Only mRS score was assessed 3 months after the onset of stroke, while long-term neurological and cognitive outcomes of NCSE remain unknown. (3) Our study did not explore the impact of the duration of NCSE and treatment therapy on outcomes; the clinical implications of NCSE in stroke patients remain controversial because the exact duration of epileptiform discharge is difficult to determine in some patients because of the subtlety of the clinical features. (4) We did not consider the effects of life-sustaining therapy on patient prognosis. Given that maintenance treatment may affect the prognosis of patients as an external factor, this may have an impact on the final results to some extent. Despite these limitations, our study has significant implications. This is the first prospective clinical study of NCSE in severe stroke patients presenting with altered consciousness, and the results indicate that it is an independent predictor of unfavorable functional outcomes, which provides strong support for further investigations into the influence of antiepileptic treatments on outcomes. Furthermore, we explored the prevalence of NCSE and identified the risk factors for its occurrence.

## Conclusion

These results suggest that NCSE is an EEG pattern commonly seen in the acute phase of stroke. Cortical involvement and decreased level of consciousness should raise suspicion for this condition, and at least 24 h of video-EEG monitoring of these patients can help in its diagnosis. Our study also suggests that the occurrence of NCSE may have a negative effect on the patients and lead to unfavorable outcomes, which provides strong support for further investigations into the influence of antiepileptic treatments on outcomes.

## Data Availability Statement

The original contributions presented in the study are included in the article/supplementary material, further inquiries can be directed to the corresponding authors.

## Ethics Statement

The studies involving human participants were reviewed and approved by Ethics Committee of the 6th People's Hospital of Shanghai, School of Medicine, Shanghai Jiaotong University, Shanghai, China. Written informed consent to participate in this study was provided by the participants' legal guardian/next of kin.

## Author Contributions

LZ conceived the project. LZ and WZ designed the experiments, carried out the majority of the experiments, and wrote the manuscript. FC participated in the discussion and proposed helpful suggestions. XB and LX collected data and analyzed data. ML and ZG analyzed data and proofread the manuscript. All authors contributed to the article and approved the submitted version.

## Conflict of Interest

The authors declare that the research was conducted in the absence of any commercial or financial relationships that could be construed as a potential conflict of interest.

## Publisher's Note

All claims expressed in this article are solely those of the authors and do not necessarily represent those of their affiliated organizations, or those of the publisher, the editors and the reviewers. Any product that may be evaluated in this article, or claim that may be made by its manufacturer, is not guaranteed or endorsed by the publisher.

## References

[1] Neurocritical Care Committee of the Chinese Society of N. Recommendations for electroencephalography monitoring in neurocritical care units. Chin Med J. (2017) 130:1851–5. 10.4103/0366-6999.211559 28748859 PMC5547838

[2] Herman ST Abend NS Bleck TP Chapman KE Drislane FW Emerson RG. Consensus statement on continuous EEG in critically ill adults and children, part I: indications. J Clin Neurophysiol. (2015) 32:87–95. 10.1097/WNP.0000000000000166 25626778 PMC4435533

[3] Rossetti AO Hirsch LJ Drislane FW. Nonconvulsive seizures and nonconvulsive status epilepticus in the neuro ICU should or should not be treated aggressively: a debate. Clin Neurophysiol Pract. (2019) 4:170–7. 10.1016/j.cnp.2019.07.001 31886441 PMC6921236

[4] Powers WJ Rabinstein AA Ackerson T Adeoye OM Bambakidis NC Becker K. Guidelines for the early management of patients with acute ischemic stroke: 2019 update to the 2018 guidelines for the early management of acute ischemic stroke: a guideline for healthcare professionals from the american heart association/american stroke association. Stroke. (2019) 50:e344–418. 10.1161/STR.0000000000000211 31662037

[5] Fernandez-Torre JL Kaplan PW Hernandez-Hernandez MA. New understanding of nonconvulsive status epilepticus in adults: treatments and challenges. Expert Rev Neurother. (2015) 15:1455–73. 10.1586/14737175.2015.1115719 26559043

[6] Banoczi W. ICU-cEEG monitoring. Neurodiagn J. (2020) 60:231–71. 10.1080/21646821.2020.1824982 33207129

[7] Beniczky S Hirsch LJ Kaplan PW Pressler R Bauer G Aurlien H. Unified EEG terminology and criteria for nonconvulsive status epilepticus. Epilepsia. (2013) 54:28–9. 10.1111/epi.12270 24001066

[8] Bentes C Martins H Peralta AR Casimiro C Morgado C Franco AC. Post-stroke seizures are clinically underestimated. J Neurol. (2017) 264:1978–85. 10.1007/s00415-017-8586-9 28808783

[9] Altindag E Okudan ZV Tavukcu Ozkan S Krespi Y Baykan B. Electroencephalographic patterns recorded by continuous EEG monitoring in patients with change of consciousness in the neurological intensive care unit. Noro Psikiyatr Ars. (2017) 54:168–74. 10.5152/npa.2016.14822 28680316 PMC5491668

[10] Singh J Thakur G Alexander J Rayi A Peng J Bell W. Predictors of nonconvulsive seizure and their effect on short-term outcome. J Clin Neurophysiol. (2021) 38:221–5. 10.1097/WNP.0000000000000687 32141985

[11] Miyaji Y Kawabata Y Joki H Seki S Mori K Kamide T. Late seizures after stroke in clinical practice: the prevalence of non-convulsive seizures. Intern Med. (2017) 56:627–30. 10.2169/internalmedicine.56.7162 28321060 PMC5410470

[12] Egawa S Hifumi T Kawakita K Manabe A Nakashima R Matsumura H. Clinical characteristics of non-convulsive status epilepticus diagnosed by simplified continuous electroencephalogram monitoring at an emergency intensive care unit. Acute Med Surg. (2017) 4:31–7. 10.1002/ams2.221 29123833 PMC5667301

[13] Lima FO Ricardo JAG Coan AC Soriano DC Avelar WM Min LL. electroencephalography patterns and prognosis in acute ischemic stroke. Cerebrovasc Dis. (2017) 44:128–34. 10.1159/000477674 28605741

[14] Dupont S Kinugawa K. Nonconvulsive status epilepticus in the elderly. Rev Neurol. (2020) 176:701–9. 10.1016/j.neurol.2019.12.007 32169326

[15] Santa Cruz R Villarejo F Figueroa A Cortes-Jofre M Gagliardi J Navarrete M. Mortality in critically ill elderly individuals receiving mechanical ventilation. Respir Care. (2019) 64:473–83. 10.4187/respcare.06586 30944228

[16] Scoppettuolo P Gaspard N Depondt C Legros B Ligot N Naeije G. Epileptic activity in neurological deterioration after ischemic stroke, a continuous EEG study. Clin Neurophysiol. (2019) 130:2282–6. 10.1016/j.clinph.2019.09.005 31594733

[17] Scholtes FB Renier WO Meinardi H. Non-convulsive status epilepticus: causes, treatment, and outcome in 65 patients. J Neurol Neurosurg Psychiatry. (1996) 61:93–5. 10.1136/jnnp.61.1.93 8676169 PMC486466

[18] Ferrari-Marinho T Perucca P Amiri M Dubeau F Gotman J Caboclo LO. High-frequency oscillations in the scalp eeg of intensive care unit patients with altered level of consciousness. J Clin Neurophysiol. (2020) 37:246–52. 10.1097/WNP.0000000000000624 31365358

[19] Baker AM Yasavolian MA Arandi NR. Nonconvulsive status epilepticus: overlooked and undertreated. Emerg Med Pract. (2019) 21:1–24. 31557430

[20] Mader EC Jr Losada V Baity JC McKinnies EM Branch LA. Stroke-onset seizures during midbrain infarction in a patient with top of the basilar syndrome. J Investig Med High Impact Case Rep. (2020) 8:1–11. 10.1177/2324709620940497 32646241 PMC7357055

[21] Kramer A Kromm J. What is the role of continuous electroencephalography in acute ischemic stroke and the relevance of the “ictal-interictal continuum”? Neurocrit Care. (2020) 32:687–90. 10.1007/s12028-020-00945-z 32246436

[22] Lee DY Jiu YR Hsieh CL. Electroacupuncture at zusanli and at neiguan characterized point specificity in the brain by metabolomic analysis. Sci Rep. (2020) 10:10717. 10.1038/s41598-020-67766-0 32612281 PMC7329888

[23] Chen WC Magill ST Englot DJ Baal JD Wagle S Rick JW. Factors associated with pre- and postoperative seizures in 1033 patients undergoing supratentorial meningioma resection. Neurosurgery. (2017) 81:297–306. 10.1093/neuros/nyx001 28327947 PMC5572484

[24] Wang Z Tian C Dhamala M Liu Z. A small change in neuronal network topology can induce explosive synchronization transition and activity propagation in the entire network. Sci Rep. (2017) 7:561. 10.1038/s41598-017-00697-5 28373712 PMC5428839

[25] Tomari S Tanaka T Matsubara S Fukuma K Ihara M Nagatsuka K. Risk factors for nonconvulsive status epilepticus after stroke. Eur Neurol. (2018) 80:256–60. 10.1159/000496512 30716731

[26] Belcastro V Vidale S Gorgone G Pisani LR Sironi L Arnaboldi M. Non-convulsive status epilepticus after ischemic stroke: a hospital-based stroke cohort study. J Neurol. (2014) 261:2136–42. 10.1007/s00415-014-7471-z 25138478

[27] Matsubara S Sato S Kodama T Egawa S Nakamoto H Toyoda K. Nonconvulsive status epilepticus in acute intracerebral hemorrhage. Stroke. (2018) 49:1759–61. 10.1161/STROKEAHA.118.021414 29880553

[28] Young GB Jordan KG Doig GS. An assessment of nonconvulsive seizures in the intensive care unit using continuous EEG monitoring: an investigation of variables associated with mortality. Neurology. (1996) 47:83–9. 10.1212/WNL.47.1.83 8710130

[29] Silveira DC Sagi A Romero R. Are seizures predictors of mortality in critically ill patients in the intensive care unit (ICU)? Seizure. (2019) 73:14–6. 10.1016/j.seizure.2019.10.009 31689583

[30] Tabaeizadeh M Aboul Nour H Shoukat M Sun H Jin J Javed F. Burden of epileptiform activity predicts discharge neurologic outcomes in severe acute ischemic stroke. Neurocrit Care. (2020) 32:697–706. 10.1007/s12028-020-00944-0 32246435 PMC7416505

[31] DeLorenzo RJ Waterhouse EJ Towne AR Boggs JG Ko D DeLorenzo GA. Persistent nonconvulsive status epilepticus after the control of convulsive status epilepticus. Epilepsia. (1998) 39:833–40. 10.1111/j.1528-1157.1998.tb01177.x 9701373

[32] Tsai MH Chuang YC Chang HW Chang WN Lai SL Huang CR. Factors predictive of outcome in patients with de novo status epilepticus. QJM. (2009) 102:57–62. 10.1093/qjmed/hcn149 19015144

[33] Shneker BF Fountain NB. Assessment of acute morbidity and mortality in nonconvulsive status epilepticus. Neurology. (2003) 61:1066–73. 10.1212/01.WNL.0000082653.40257.0B 14581666

[34] Kikuta Y Kubota Y Nakamoto H Chernov M Kawamata T. Nonconvulsive status epilepticus after surgery for ruptured intracranial aneurysms: incidence, associated factors, and impact on the outcome. Clin Neurol Neurosurg. (2021) 200:106298. 10.1016/j.clineuro.2020.106298 33268192

[35] Vespa PM Miller C McArthur D Eliseo M Etchepare M Hirt D. Nonconvulsive electrographic seizures after traumatic brain injury result in a delayed, prolonged increase in intracranial pressure and metabolic crisis. Critical Care Medicine. (2007) 35:2830–6. 10.1097/01.CCM.0000295667.66853.BC 18074483 PMC4347945

[36] Struck AF Westover MB Hall LT Deck GM Cole AJ Rosenthal ES. Metabolic correlates of the ictal-interictal continuum: FDG-PET during continuous EEG. Neurocrit Care. (2016) 24:324–31. 10.1007/s12028-016-0245-y 27169855 PMC5478419

[37] Witsch J Frey HP Schmidt JM Velazquez A Falo CM Reznik M. Electroencephalographic periodic discharges and frequency-dependent brain tissue hypoxia in acute brain injury. JAMA Neurol. (2017) 74:301–9. 10.1001/jamaneurol.2016.5325 28097330 PMC5548418

[38] De Marchis GM Pugin D Meyers E Velasquez A Suwatcharangkoon S Park S. Seizure burden in subarachnoid hemorrhage associated with functional and cognitive outcome. Neurology. (2016) 86:253–60. 10.1212/WNL.0000000000002281 26701381 PMC4733156

[39] Zafar SF Postma EN Biswal S Boyle EJ Bechek S O'Connor K. Effect of epileptiform abnormality burden on neurologic outcome and antiepileptic drug management after subarachnoid hemorrhage. Clin Neurophysiol. (2018) 129:2219–27. 10.1016/j.clinph.2018.08.015 30212805 PMC6478499

[40] Cuomo O Rispoli V Leo A Politi GB Vinciguerra A di Renzo G. The antiepileptic drug levetiracetam suppresses non-convulsive seizure activity and reduces ischemic brain damage in rats subjected to permanent middle cerebral artery occlusion. PLoS ONE. (2013) 8:e80852. 10.1371/journal.pone.0080852 24236205 PMC3827478

[41] DeGiorgio CM Heck CN Rabinowicz AL Gott PS Smith T Correale J. Serum neuron-specific enolase in the major subtypes of status epilepticus. Neurology. (1999) 52:746–9. 10.1212/WNL.52.4.746 10078721

[42] Bongiovanni F Romagnosi F Barbella G Di Rocco A Rossetti AO Taccone FS. Standardized EEG analysis to reduce the uncertainty of outcome prognostication after cardiac arrest. Intensive Care Med. (2020) 46:963–72. 10.1007/s00134-019-05921-6 32016534

[43] Lee JJ Park KI Park JM Kang K Kwon O Lee WW. Clinical characteristics and treatment outcomes of de novo nonconvulsive status epilepticus: a retrospective study. J Clin Neurol. (2021) 17:26–32. 10.3988/jcn.2021.17.1.26 33480195 PMC7840313

[44] Onder H Arsava EM Topcuoglu MA Dericioglu N. Do video-EEG monitoring findings in ICU patients with acute stroke predict development of seizures and survival during follow-up? Clin EEG Neurosci. (2017) 48:417–21. 10.1177/1550059417727225 28844159

